# Assessment of the environmental kuznets curve within EU-27: Steps toward environmental sustainability (1990–2019)

**DOI:** 10.1016/j.ese.2023.100312

**Published:** 2023-09-19

**Authors:** Safwan Mohammed, Abid Rashid Gill, Kaushik Ghosal, Main Al-Dalahmeh, Karam Alsafadi, Szilárd Szabó, Judit Oláh, Ali Alkerdi, Akasairi Ocwa, Endre Harsanyi

**Affiliations:** a Institute of Land Use, Engineering and Precision Farming Technology, Faculty of Agricultural and Food Sciences and Environmental Management, University of Debrecen, Böszörményi 138, H-4032, Debrecen, Hungary; b Institutes for Agricultural Research and Educational Farm, University of Debrecen, Böszörményi 138, H-4032, Debrecen, Hungary; c Department of Economics, The Islamia University of Bahawalpur, Pakistan; d Department of Mining Engineering, Indian Institute of Engineering Science and Technology, Shibpur, P.O. 711103, Botanic Garden, Howrah, West Bengal, 711103, India; e Institute of Management and Organization Sciences, Faculty of Economics and Business, University of Debrecen, Egyetem Tér 1, 4032, Debrecen, Hungary; f School of Geographical Sciences, Nanjing University of Information Science and Technology, Nanjing, 210044, China; g Department of Physical Geography and Geoinformatics, Faculty of Sciences and Technology, University of Debrecen, Egyetem tér 1, 4032, Debrecen, Hungary; h Faculty of Economics and Business, University of Debrecen, 4032, Debrecen, Hungary; i Department of Trade and Finance, Faculty of Economics and Management, Czech University of Life Sciences, Prague, 16500, Prague, Czech Republic; j Department of Agricultural Economics, Faculty of Agriculture, Kahramanmaras Sütçü Imam University, Turkey; k Department of Agriculture Production, Faculty of Agriculture, Kyambogo University P.O.B. 1, Kyambogo, Kampala, Uganda

**Keywords:** Climate change, Environmental management, Sustainable communities, Green growth

## Abstract

Reducing environmental pollution is a critical goal in global environmental economics and economic development. The European Union (EU) faces environmental challenges due to its development activities. Here we present a comprehensive approach to assess the impact of carbon dioxide (CO_2_) emissions, energy consumption (EC), population structure (POP), economy (GDP), and policies on the environment within the EU using the environmental Kuznets curve (EKC). Our research reveals that between 1990 and 2019, the EU-27 experienced an increase of +1.18 million tonnes of oil equivalent (Mtoe) per year in energy consumption (*p* < 0.05), while CO_2_ emissions decreased by 24.25 million tonnes (Mt) per year (*p* < 0.05). The highest reduction in CO_2_ emissions occurred in Germany (−7.52 Mt CO_2_ annually), and the lowest in Latvia (−0.087 Mt CO_2_ annually). The empirical EKC analysis shows an inverted-U shaped relationship between GDP and CO_2_ emissions in the EU-27. Specifically, a 1% increase in GDP results in a 0.705% increase in carbon emission, while a 1% increase in GDP^2^ leads to a 0.062% reduction in environmental pollution in the long run (*p* < 0.01). These findings indicate that economic development within the EU has reached a stage where economic growth positively impacts the environment. Overall, this study provides insights into the effectiveness of environmental policies in mitigating degradation and promoting green growth in the EU 27 countries.

## Highlights

• The total GHG emissions of the EU-27 were reduced significantly (*p* < 0.05).
• The energy consumption increased significantly by +1.18 Mtoe per year (*p* < 0.05).
• The Environmental Kuznets Curve relation has been confirmed within EU-27.
• Economic development within the EU-27 has started to improve the environment.

## 1 Introduction

The global economy has experienced remarkable growth in the past century due to various factors, such as globalization, industry booms, innovation, significant technological development, free international trade at multiple levels and sectors, and the overall economic environment. Nevertheless, this economic progress has adversely affected the environment, leading to heightened environmental pollution and intensified environmental degradation [1]. Economic activities have played a crucial role in promoting development and growth but have also contributed to the emission of greenhouse gases (GHGs), such as carbon dioxide (CO_2_), methane (CH_4_), and nitrous oxide (N_2_O). These GHGs have contributed to environmental changes, ecosystem degradation, and rapid climate change [2,3]. Various factors influence GHG emissions, including population increase, per capita consumption and output growth, infrastructure choices, innovation, technology, and human behavior [[4], [5], [6]]. However, if mitigation measures are insufficient, economic growth and human and social development potentials will likely decline because of climate change [7].

Environmental degradation remains a main concern in the European Union (EU). Therefore, any operations that involve rising energy consumption (EC) and the exhaustion of carbon (CO_2_) have effects on the environment [8,9]. On the other hand, production and consumption activities are linked to energy use; thus, energy is critical for economic growth. Consequently, the EU's foundation for economic activities and social well-being is secure [10]. The EU's historical reliance on fossil fuels as an energy source has notably escalated the emission of potentially hazardous gases [11]. In June 2021, EU-27 countries set the European Green Deal under the auspices of European Climate Law (2021/1119) to reduce GHGs, aiming at ‘net-zero’ emissions by 2050 [12]. The law's primary goal is to decrease GHG emissions by 55% by 2030 compared to 1990 (‘Fit for 55’) [13]. The EU has targeted reducing CO_2_ emissions by 310 million tons using natural sinks, demonstrating its leadership in pursuing carbon neutrality. The ‘Fit for 55’ initiative is the most important regulation package to reach the targets of the Paris Agreement, keeping global warming below 1.5 °C. At its establishment, acceptance of ‘Fit for 55′ was crucial. If a robust framework exists, the EU stands a decent chance of leading the global fight to attain net-zero emissions.

The EU countries have implemented policies related to climate change mitigation, energy security, energy efficiency, use of renewable energy, and others to curb environmental degradation and recorded considerable progress. This article expands the existing literature by providing a holistic approach to evaluating the impact of energy consumption, population structure, economy, and policies on the environmental sector across the EU through the lens of the environmental Kuznets curve (EKC).

The main aims of this study were to (1) comprehensively report on the contemporary changes (trends) in GHG emissions and energy consumption across the EU-27 between 1990 and 2019 and (2) highlight the interaction between GHG emissions, energy consumption, population, and gross domestic product (GDP) by employing the EKC. The study's main focus was to address a crucial question: the effectiveness of EU-27 environmental policies in mitigating environmental degradation. To accomplish these goals, the article is organized into four sections: (1) literature review, which presents a comprehensive overview of the relevant literature, (2) material and method, (3) findings of this research, (4) discussion of research output concerning the research limitations.

## 2 Literature review

Climate change and global warming are among the worst externalities exclusively being debated by scientists, policymakers, governments, and societies [14]. The primary focus is on the escalating GHG levels, especially CO_2_ emissions, which account for 60% of GHGs [15]. Increased GHG emissions reduce environmental quality by increasing environmental pollution [14], which is detrimental to sustainable development [16]. Nations are currently grappling with twin challenges of achieving and maintaining economic growth and environmental quality [17,18] exacerbated by globalization trade openness, urbanization development, and prosperity of the service sector, causing relevant structural changes in trade, energy, economy, and society [19]. Hence, decreasing pollution has become a significant concept in the environmental economics and economic development model [7]. To understand this nexus, economist Simon Kuznets introduced the EKC in 1955 [20], later formalized by Grossman and Krueger [21]. The EKC indicates that, initially, environmental degradation is caused by economic growth in the first production stage. In addition, the EKC illustrates the probable relationship between economic growth and rising income levels instead of the effect on economic inequality, demonstrating that economic disparity rises and falls as the economy grows [22]. However, the tendency may be inverse; it may decouple the effects of progress on the environment because economic progress can lead to environmental improvements and a virtual reduction in per capita emissions of national production. This relationship is nonlinear between emissions per capita or unit of GDP and wealth [23]. Therefore, EKC serves as a metaphor for understanding the fundamental mechanisms of public policy interventions that reduce economic growth from harming the environment [24].

Several studies have analyzed the nexus between the environment and economic performance indicators such as GDP, trade, urbanization, and others at national, regional, and global levels (Table 1). Many empirical investigations have yet to reach consistent conclusions on the connection between CO_2_ and GDP [18] since studies select different input variables, time, and regional scopes.

**Table 1:** Survey of econometric literature of EKC implementation showing the nexus between environmental indicators and carbon emissions from a global perspective.

Country	Period	Sector	GHGs	Input variable	Output	Reference
208 countries	1990–2018	Industry	CO_2_	Renewable energy (RE), GDP, trade, human capital index, natural resources rents	Income level and CO_2_ emissions had EKC relation	[18]
PIIGS countries (Portugal, Ireland, Italy, Greece, and Spain)	1990–2019	Industry	CO_2_	Economic complexity index (ECI), foreign direct investment, RE, urbanization process	ECI. and CO_2_ emissions had inverted-U and further *N*-shaped EKC	[14]
Seven Countries (Emerging industrialized economy)	1995–2016	Industry	CO_2_	GDP, RE, institutional quality	EKC validated. Renewable energy reduces pollution. Weak institutions reduce environmental quality	[87]
18 Emerging economies/countries	1990–2015	Industry	CO_2_	Trade openness RE, non- RE, GDP, GDP^2^.	RE affects CO_2_ emissions negatively and reverses renewable energy	[88]
BRICS countries	1995–2014	Industry	CO_2_	EC, financial development, GDP, GDP^2^, and globalization urbanization.	GDP and GDP^2^ had negative and positive effects (valid EKC)	[27]
26 E U. countries	1995–2018	Industry	CO_2_	ECI, Brexit, and other crisis episodes	Tourism, energy use, real GDP per capita increased emissions. In either scenario EKC hypothesis	[6]
47 countries	1990–2014	Industry	CO_2_	Government consumption expenditure, government expenditures, GDP, GDP^2^, EC, financial development, globalization, and institutional quality	EKC confirmed. EC, financial development, and globalization increased CO_2_ emissions	[31]
India	1971–2015	Industry	CO_2_	GDP and RE	Inverted U-shaped EKC Renewable energy has a significant negative effect on CO_2_ emissions.	[30]
25 OECD countries	1980–2010	Industry (energy and trade sector)	CO_2_	Trade, RE, GDP, and non-RE consumption	Inverted U-shaped EKC. Increased non- RE increased CO_2_ emission.	[29]
134 countries	1996–2015	Industry	CO_2_	Trade openness, population aging, RE, and natural resource rent	Inverted U-shape EKC in lower-middle-income countries. Renewable energy and population aging improve the environment	[33]
Ten ASEAN countries	1993–2014	Industry	CO_2_	GDP, foreign investment, trade openness index, and urbanization	Earlier inverted U-shaped and a later *N*-shaped EKC	[25]
27 E U. countries	1995–2017	Industry	CO_2_	Economic complexity Index, energy intensity or consumption	Inverted U-shaped curve. 10% rise in energy intensity increased CO_2_ emissions by 3.9%	[37]
Venezuela	1971–2013	Industry	CO_2_	GDP, GDP^2^, EC, financial development, and government expenditure	EKC confirmed. Government expenditure positively impacted on environmental degradation.	[35]
Five ASEAN countries	1971–2014	Industry	CO_2_	GDP, financial development, EC, and globalization urbanization	EKC hypothesis supported. All variables increased CO_2_ emissions except per capita GDP	[28]
Malaysia	1970–2016	Industry	CO_2_	Natural resources (GDP)	Conventional EKC pattern not supported.	[89]
16 lower income, 26 lower middle income, 26 higher middle income, and 31 high-income countries	1980–2008	Industry	CO_2_	Trade openness, financial development, GDP, EC, and urbanization,	Inverted U-shaped nexus between ecological footprint and GDP in high- and middle-income countries	[34]
56 countries	2003–2018	Industry	CO_2_	Income inequality	Income inequality had a double-threshold effect on the economic growth and carbon per capita emission.	[17]

The findings of several studies exploring the relationship between carbon emissions and economic growth in various countries and regions have been documented in previous research. Le [25] surveyed across ten Association of Southeast Asian Nations (ASEAN) countries and revealed a U-shaped EKC in the early development stages, which later transformed into an *N*-shaped EKC hypothesis in the long run. This suggests negative and positive causality between carbon emissions and economic growth. Yuelan et al. [26] studied between 1980 and 2016 in China, revealed that energy usage, GDP, total revenue, and government expenditures deteriorated environmental quality. Analysis of the BRICS countries (i.e., Brazil, Russia, India, and China) between 1995 and 2014 showed that the GDP^2^ and GDP had negative and positive effects (valid EKC), while urbanization and globalization have a negative insignificant relationship with CO_2_ emission. The report revealed a two-way causality between EC, GDP^2^, financial development, and economic growth with CO_2_ emission [27]. Phong [28] studied five ASEAN countries between 1971 and 2014 and confirmed the EKC hypothesis. Results further revealed that except per capita GDP, globalization, EC, urbanization, and financial development increased CO_2_ emissions.

The latest study by Wang et al. [18] indicated that CO_2_ emissions (per capita) nexus with income (per capita) resulted in an “inverted U-shaped” EKC after human capital, trade openness, and natural resource rents were included as variable inputs. Renewable energy consumption had a more substantial carbon emission reduction effect before the EKC turning point, while human capital was after the EKC turning point. Ben Jebli et al. [29] reported an inverted U-shaped EKC between per capita CO_2_ emissions and GDP in the 25 OECD (Organization for Economic Co-operation and Development) countries. Balsalobre-Lorente et al. [14] reported that renewable energy (RE) inhibits CO_2_ emissions while urbanization exerts vast pressure on environmental quality. Sinha and Shahbaz [30] in India, covering a period between 1971 and 2015, reported an inverted U-shaped EKC. The study revealed that RE had a significant adverse effect on CO_2_ emissions. Le and Ozturk [31] studied 47 developing and market economies between 1990 and 2014 and confirmed the inverted U-shaped EKC; however, financial development, globalization, and energy consumption increased CO_2_ emissions. In Vietnam, industrialization, per capita GDP, and EC raised CO_2_ emissions, while globalization lowered CO_2_ emissions in the long run [32]. Meanwhile, Wang et al. [33] also reported RE and population aging to improve environmental quality.

According to the research findings, income inequality had a double-threshold effect on economic growth and carbon per capita emission. It redefined the EKC hypothesis from an inverted U-shaped curve to an *N*-shaped curve depending on the income groups making decoupling carbon emission and economic growth complex [17]. Another study by Al-mulali et al. [34] found an inverted U-shaped EKC between ecological footprint and GDP in the economic development stage of high- and middle-income countries with better energy saving, efficiency, and use of renewable energy. Therefore, improving financial investments targeting the reduction of carbon emissions is vital since financial investments reduce environmental damage [34]. Financial development enhanced environmental quality while government expenditure increased the pollution level in Venezuela [35]. Therefore, comprehensive energy and economic policies targeting cleaner production should be advocated due to the positive nexus between economic complexity and environmental quality [14]. A robust and efficient financial sector can play a role in environmental protection by promoting environmentally friendly and energy-efficient projects and advancing loans for businesses with conditions of reducing CO_2_ emissions [27].

**Fig. 1:**
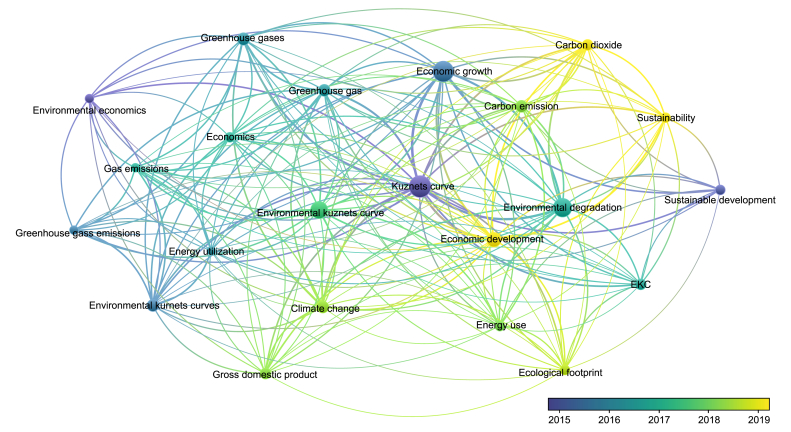
Bibliographic analysis using Vosviewer software exhibits the relationship between climate change, environmental degradation, economic development, GDP, GHGs, energy use, and sustainability.

The reviewed literature indicates the relationship between CO_2_ emissions GDP, income inequality, renewable energy consumption, and CO_2_ emissions within the EKC hypothesis, wherein CO_2_ was an indicator of environmental harm. Although most studies have focused on these variables, only one study included the population as a variable input. By employing the Vos viewer for bibliographic analysis [36], recent research between 2018 and 2019 focused on understanding the relationship between climate change, environmental degradation, economic development, gross domestic product, energy use, and sustainability using EKC (Fig. 1). In this context, a study by Balsalobre-Lorente et al. [14] confirmed the existence of an inverted U- and later *N*-shaped EKC nexus between CO_2_ emissions and economic complexity, foreign direct investment, renewable energy, and urbanization process in PIIGS countries (i.e., Portugal, Italy, Ireland, Greece, and Spain) in the period of 1990–2019. Accordingly, in the 25 EU countries from 1995 to 2017, a relationship between CO_2_ emissions and economic complexity produced an inverted U-shaped EKC with a 10% rise in energy intensity, increasing CO_2_ emissions by 3.9% [37]. On the other hand, another study [38] explored the connection between environmental degradation and energy innovation and economic growth in 33 EU countries from 1996 to 2017. Results indicated that energy innovation negatively and significantly impacted environmental degradation, while GDP produced a U-shaped EKC. Cai et al. [5] also revealed no cointegration between the consumption of clean energy, real GDP, and CO_2_ emissions in France and Italy except in German and other non-EU countries, such as Canada and the U.S. Overall, different studies showed non-unidirectional relationship effects between economic indicators and environmental quality, emphasizing the necessity for continuous evaluation. Besides, our study differs from these by introducing population as a controlling variable input. The EU27 exhibits varying population structures, leading to different patterns in energy consumption, land resource utilization, and urbanization development. Earlier, Wang et al. [33] reported the aging population could positively impact environmental quality.

## 3 Material and method

### 3.1 Data collection and trend analysis

Data for EU countries (1990–2019) were collected from the website of the European Commission, DG Energy, Unit A4. Data included (1) CO_2_ emissions - National total (including International Aviation, without Land Use, Land-Use Change, and Forestry (LULUCF)) (Mt CO_2_), (2) energy consumption (Mtoe), (3) GDP-market prices (Mrd EUR at current prices), and (4) population (thousands of people).

To capture the trend and magnitude of change in each variable, the Mann–Kendall test (*MK*_*t*_) [39] and Sen's slope estimator (ρ) [40] were employed. *MK*_*t*_ has been widely used in trend analysis, and it is calculated as shown in equation (1):

(1) $${MK}_{t}=\sum_{k=1}^{n-1}\sum_{j=k+1}^{n}\text{sign}\left(x_{j}-x_{k}\right)$$

where $x_{j},x_{k}$ are the values for the studied variable in years *j* and *k* (*j* > *k*); n is the sum of the years; and $\text{sign}\left(x_{j}-x_{k}\right)$ is the sign function.

The variance of equation (1) and ($\nabla_{{MK}_{t}}$) can be denoted as equation (2):

(2) $$\nabla_{{MK}_{t}}=\frac{n\left(n-1\right)\left(2n+5\right)-\sum_{i=1}^{m}t_{i}\left(t_{i}-1\right)\left(2t_{i}+5\right)}{18}$$

However, the Z statistic (*Z*) can be estimated as equation (3):

(3) $$Z=\left\{ \begin{array}{c} \frac{\left({MK}_{t}\right)-1}{\sqrt{\nabla_{{MK}_{t}}}},\text{if}\;{MK}_{t}>0\\ 0,\text{if}\;{MK}_{t}=0\\ \frac{\left({MK}_{t}\right)+1}{\sqrt{\nabla_{{MK}_{t}}}},\text{if}\;{MK}_{t}<0 \end{array}\right.$$

The *p*-value for equation (3) is estimated based on equation (4):

(4) $$p=0.5-\frac{1}{\sqrt{\pi }}\int_{0}^{\left|Z\right|}e^{\frac{{-t}^{2}}{2}}dt$$

The magnitudes of the studied variables are captured using Sen's slope (Ss) [40] in equation (5):

(5) $$Ss=\frac{x_{j}-x_{k}}{j-k}\;\text{for}\;i=1,2,3,\ldots ,n$$

In this case, Sen's slope (Ss) (ρ) is the median of the time series (Г) for *n* values and is denoted in equation (6):

(6) $$\rho =\left\{ \begin{array}{l} {Г}_{\frac{n+1}{2}},\;n\;\text{is}\;\text{odd}\\ \frac{1}{2}\left({Г}_{\frac{n}{2}}+{Г}_{\frac{n+2}{2}}\right),\;n\;\text{is}\;\text{even} \end{array}\right.$$

where a positive value of ρ indicates an increasing trend, while a negative value refers to a declining tendency, finally, the results were visualized using the geographical information system (GIS).

### 3.2 Trend change point detection

To identify the trend-changing points (years) in the time series, a homogeneity nonparametric Buishand range (BR) [41] test was applied. The BR test is based on the null hypothesis assumption that the variables are independent of each other with a normal distribution, while the alternate hypothesis assumes a shift in the mean of the time series. It is probably sensitive to find breaks in the middle of the time series. In the BR test, rescale adjusted partial sums ($S_{k})$ are computed by:

(7) $$S_{k}=\sum_{i=1}^{k}\left(X_{i}-\bar{X}\right)\;k=1,2,3,\ldots ,n$$

where $X_{i}$ represents the observed data series, $\bar{X}$ represents the mean, and n represents the total data numbers in the time series [42]. The value of $S_{k}$ rotates around zero for a homogeneous time series. If there is a break in year *k*, $S_{k}$ reaches the maximum negative of the maximum positive shift [43]. The equation computes significant change shift:

(8) $$R=\frac{\max_{s_{k}}-\min_{s_{k}}}{\bar{X}}$$

### 3.3 The EKC analysis

The legitimacy of the EKC in 27 European countries has been examined based on the following model, which is formulated depending on different studies [44,45].

(9) $${\mathrm{LN}\left({\text{CO}}_{2}\right)}_{it}=\beta_{1}+\beta_{2}{\mathrm{LN}\left(\text{GDP}\right)}_{it}+\beta_{3}\;{\mathrm{LN}\left(\text{GDP}\right)}_{it}^{2}+\beta_{4}{\mathrm{LN}\left(\text{EC}\right)}_{it}+\beta_{5}{\mathrm{LN}\left(\text{POP}\right)}_{it}+U_{it}$$

In this equation, $\beta_{1}$ is constant; EC, POP, and *U*_*it*_ represent energy consumption, population, and the error term. The term (_it_) subscript indicates panel data: i for countries and t for the time period. The quadratic term of GDP is included to model the nonlinear relationship between GDP (income) and ${\text{CO}}_{2}\;($ the environment).

The EKC hypothesis is verified in the context of the EU-27 if the estimation of equation (9) proves that $\beta_{1}$ > 0 and $\beta_{2}<0$. Two important parameters, EC and POP, are included to run the model properly. The sign of the coefficient of energy consumption and population growth is expected to be positive [46,47]. The data about the variables under investigation covers the period from 1990 to 2019. All data were transformed into the logarithmic form to obtain the elasticities of carbon emissions for explanatory variables; therefore, coefficients were considered long-run elasticities.

The cross-sectional dependency test of Pesaran [48] is employed to ascertain whether each panel is cross-sectionally independent, where the null hypothesis is that the panels are cross-sectionally independent.

We employed a three-stage estimation method to estimate the coefficients of equation (9). In the first stage, the first and second generations of the panel unit root test were applied to examine the stationarity properties of the panel data, where the following unit root tests, Im and Pesaran [49], Levin, Lin and Chu [50], and Maddala and Wu [51], were applied. Moreover, Pesaran [52] altered the I.P.S. test by incorporating cross-sectional dependence (CIPS). However, for each panel data presenting nonstationary, the first difference was taken to make it stationary (*H*_0_: the panel data are nonstationary); thus, the cointegration relation among the variables was investigated by applying panel cointegration tests developed by Pedroni [53] and Kao [54]. Pedroni [53] developed seven test statistics based on the cointegration regression in equations (10), (11), which we used to test this null hypothesis that there is no cointegration relation among the variables in equation (9).

(10) $$y_{it}=\alpha_{it}+\delta_{it}t+\beta_{i}x_{it}+\varepsilon_{it}$$

(11) $${\Delta y}_{it}={\beta_{i}\Delta x}_{it}+n_{it}\left(n_{it}=\varepsilon_{it}-\varepsilon_{it-1}\right)$$

In equation (10), *y*_*it*_ and *x*_*it*_ are dependent and explanatory variables, respectively, while $\varepsilon_{it}$ and *α*_*it*_ indicates residual and fixed effects. In equation (11), Δ is the difference operator. Moreover, referring to Kao [54], the Pedroni cointegration relation was verified. We also employed the Westerlund [55] panel cointegration tests (Ga, Gt, Pa, and Pt) based on the error correction model (ECM), as it has several advantages compared to other panel cointegration methodologies.

Firstly, it enables heterogeneity in short- and long-run dynamics. Secondly, it overcomes the problem of cross-sectional dependency. Lastly, this approach relies upon the bootstrap procedure that includes repeating various cointegration methods [56].

The long-run co-integration relation between the dependent and explanatory variables of equation (9) was also estimated. The dynamic ordinary least squares (DOLS) and fully modified ordinary least squares (FMOLS) panel cointegration estimates developed by Pedroni [57] are commonly used in literature. These estimates eliminate the endogeneity and autocorrelation problem and therefore produce efficient estimates.

However, if the panel sections are correlated and have a cross-sectional dependency, the FMOLS and DOLS may produce inefficient estimates despite having high statistical power and robustness. Eberhardt and Teal [58] and Bond and Eberhardt [59] therefore proposed a new panel cointegration estimation method called augmented mean group (AMG) that includes a common dynamic process to account for cross-sectional dependency in panel data through a two-stage regression method as in equation (12). In this context, cross-sectional dependence is a common phenomenon of panel data resulting from policy shocks and sociopolitical linkages among countries. These effects are heterogeneous and of different degrees, leading to inefficient estimation, especially when correlated to explanatory variables [60,61].

(12) $$\text{AMG}\;\text{Stage}\;\left(i\right)\Delta {\text{EFP}}_{\text{it}}=b_{o}\Delta X_{\text{it}}+X_{it}=2\;c_{it}\Delta D_{t}+e_{\text{it}}\;\Rightarrow \;c_{it}\equiv \mu_{it}$$

(13) $$\text{AMG}\;\text{Stage}\;\left(\text{ii}\right)\Delta {\text{EFP}}_{\text{it}}=b_{o}\Delta X_{\text{it}}+X_{it}=2\;c_{it}\Delta D_{t}+{\text{e}}_{\text{it}}\;\Rightarrow \;c_{it}\equiv \mu_{it}$$

In equations (12), (13), Δ *X*, *b*, and *D* represent difference operators, explanatory variables, coefficients of explanatory variables, and dummy variables. Dummy variables capture the impact of time, while $c_{it}$ is the coefficients on the dummy variables, $\mu_{it}$ is an error term, *t* is time, and *i* is cross sections in the panel data regression. Compared to other panel estimation methods, AMG considers cross-sectional dependencies and heterogeneity issues in estimation, producing more efficient estimates [60,62].

The first stage equation represents a standard Ordinary Least Squares (OLS) regression in the first difference with dummies in the first differences ΔDt, from which we collect the estimated coefficients, which are relabeled as $\mu_{it}$. Because nonstationary variables and unobservables can substantially distort results in pooled levels regressions, this process is extracted from the pooled regression in first differences. In the second stage, this constructed variable $\mu_{it}$ is included in each of the N group-specific regressions, which also include linear trend terms to capture omitted idiosyncratic processes that evolve linearly over time. Alternatively, we can subtract $\mu_{it}$ from the dependent variable, implying that the common process is imposed on each group with a unit coefficient. In either case, the AMG estimates are derived as averages of the individual $b_{o}$ estimates, following the Mean Group approach proposed by Pearson and Smith [63].

## 4 Results

### 4.1 Trend analysis of EKC parameters within the EU-27 (1990–2019)

**Fig. 2:**
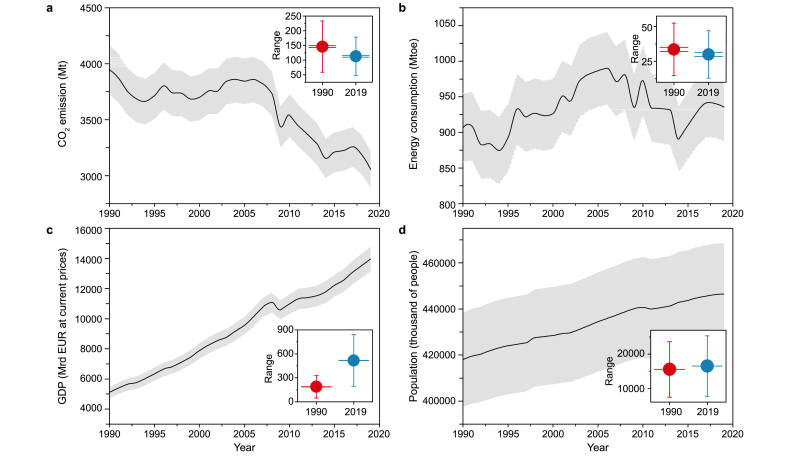
An overview of temporal trends of the studied variables (CO_2_ emissions, energy consumption, GDP, population) for all EU-27 countries (total) between 1990 and 2019 along with box plot (I-shaped box): blue color represents 2019, red color represents 1990 (•: mean; ꟾ: mean ± 95% confidence interval; gray shade around the black line represents the standard deviation between E.U. countries for each year from 1990 to 2019).

**Fig. 3:**
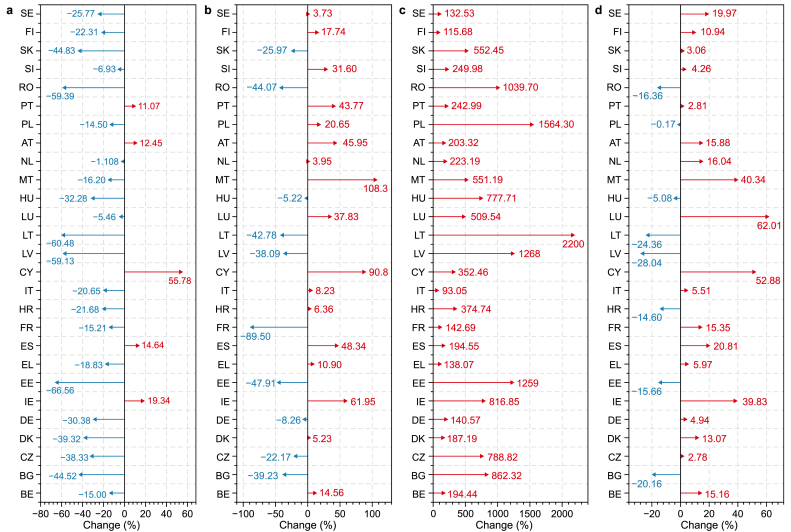
Changes (%) in EU-27 studied variables between 1990 and 2019: **a**, CO_2_ emissions; **b**, energy consumption; **c**, GDP; **d**, population. Blue arrow: decreasing trend; red arrow: increasing trend ($\text{Changes}\;\left(\%\right)=\frac{{Value\;of\;studied\;variables}_{2019}-{Value\;of\;studied\;variables}_{1990}}{{Value\;of\;studied\;variables}_{1990}}\times 100$) (Abbreviation for the Member States of the European Union (EU) as suggested by: https://ec.europa.eu/eurostat/statistics-explained/index.php?title=Glossary:Country_codes). Abbreviations: Belgium (BE), Bulgaria (BG), Czechia (CZ), Denmark (DK), Germany (DE), Estonia (EE), Ireland (IE), Greece (EL), Spain (ES), France (FR), Croatia (HR), Italy (IT), Cyprus (CY), Latvia (LV), Lithuania (LT), Luxembourg (LU), Hungary (HU), Malta (MT), Netherlands (NL), Austria (AT), Poland (PL), Portugal (PT), Romania (RO), Slovenia (SI), Slovakia (SK), Finland (FI), Sweden (SE).

Between 1990 and 2019, the EU-27 countries experienced a decrease in CO_2_ emissions alongside increases in GDP and population (Fig. 2). In this context, the total CO_2_ emission declined from 3945.0 Mt CO_2_ in 1990–3054.7 Mt CO_2_ in 2019 (Fig. 2). The EC fluctuated between 906.56 Mtoe in 1990 and 935.50 Mtoe in 2019, where the highest value was 990.13 Mtoe in 2006 (Fig. 2). Most countries exhibited a notable decrease in CO_2_ emission and an increase in GDP (Fig. 3).

**Fig. 4:**
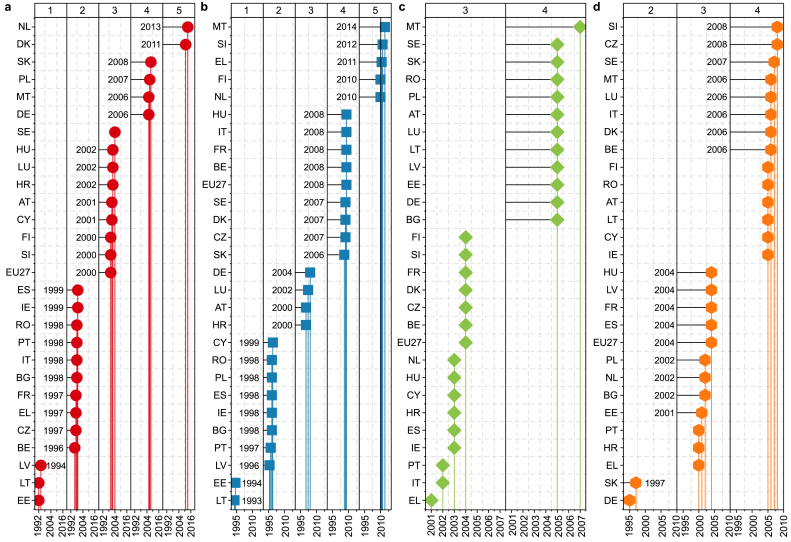
Changing years in the time series (1990–2019) in EU-27 countries for studied variables based on the BR test: **a**, energy consumption; **b**, CO_2_ emissions; **c**, GDP; **d**, population. 1: changes between 1990 and 1994; 2: changes between 1995 and 1999, 3: changes between 2000 and 2004, 4: changes between 2005 and 2010, 5: changes after 2010. Abbreviations: Belgium (BE), Bulgaria (BG), Czechia (CZ), Denmark (DK), Germany (DE), Estonia (EE), Ireland (IE), Greece (EL), Spain (ES), France (FR), Croatia (HR), Italy (IT), Cyprus (CY), Latvia (LV), Lithuania (LT), Luxembourg (LU), Hungary (HU), Malta (MT), Netherlands (NL), Austria (AT), Poland (PL), Portugal (PT), Romania (RO), Slovenia (SI), Slovakia (SK), Finland (FI), Sweden (SE).

EC increased in the EU-27 countries between 1990 and 2019 (Fig. S1a): the general trend was significant (*p* < 0.05), and the change rate was growing by +1.18 Mtoe per year. Trends of EC significantly (*p* < 0.05) increased in Spain (ES), Poland (PL), Austria (AT), Italy (IT), Ireland (IE), Portugal (PT), and Finland (FI) with positive slope and *Z* (*Ss* = 0.91–0.14, *Zs* = 3.46–2.39) with 1999, 2007, 2001, 1998, 1999, 1998, and 2000, respectively; as turning points of trend in time series (Fig. 4a). However, EC decreased in Germany (DE), Romania (RO), Czech Republic (CZ), and Sweden (S.E.) with negative slope and *Z* (*Ss* = (−0.54)–(−0.07)) (Fig. S1a). Further, the BR test identified significant turning trend years, including 2006, 1998, 1997, and 2004 for respective countries (Table S1) (Fig. 4a). The highest increase was recorded in Spain (+0.92 Mtoe; *p* < 0.05), while Germany had the largest drop (−0.55 Mtoe; *p* < 0.05).

The M.K. test showed a significant decline in CO_2_ emissions in the EU-27 (Fig. S1b). The total CO_2_ emissions from the EU-27 were reduced significantly (*p* < 0.05) by 24.25 Mt CO_2_ per year. Countries such as Germany (DE), Italy (IT), France (FR), Romania (RO), Poland (PL), Denmark (DK), and the Czech Republic (CZ) showed a significant negative (*p* < 0.05) trend with (*Ss* = (−7.4)–(−1.2)) and *Zs* = (−6.8)–(−1.8)) with significant (*p* < 0.05) trend declining year of 2004, 2008, 1998, and 2007 identified from the BR test (Fig. 4b) (Table S1). On the contrary, Spain (ES), Croatia (HR), and Portugal (PT) showed a positive but non-significant (*p* > 0.05) trend of CO_2_ emissions with a positive slope and Z-statistics (*Ss* = 1.31–0.01, *Zs* = 1.85–0.07)). Overall, the highest reduction in CO_2_ emission was recorded in Germany (DE), with −7.52 Mt CO_2_ annually, while the lowest reduction was recorded in Latvia (LV), with −0.087 Mt CO_2_ annually.

Furthermore, GDP and POP also had a positive significant (*p* < 0.05) trend (1990–2019) (Figs. S1c and d). The annual change rate in the total GDP increased by +301.56 Mrd EUR at current prices per year (*p* < 0.05), while the POP increased by +1001.3 thousand people per year (*p* < 0.05). The highest increase in the GDP was in Germany (DE) (*p* < 0.05), followed by France (*p* < 0.05), while the lowest increase was in Malta (MT) (Fig. S1c). Regarding the POP, the highest increase was in France (FR) (+342.1 thousand people per year, *p* < 0.05), with the highest *Ss* of 341 and a sharp rise since 2004 as identified from the BR test (Fig. S1d). Notably, Romania (RO) had the highest decreasing rate of −140.57 thousand people per year (*p* < 0.05) (*Ss* = −140.5 and *Zs* = −7.7) with a turning point since 2005 as identified from the BR test (Table S1) (Fig. 4d).

### 4.2 EKC analysis

#### 4.2.1 Cross-sectional dependency (CD) and Slope Homogeneity tests for EKC analysis

According to the CD and Slope Homogeneity tests, the EU-27 remained expectedly dependent on each other due to the free trade between them during the study period (*p* < 0.01; Table 2).

**Table 2:** Cross-sectional dependency (CD) in the panel data.

Test	LCO_2_	LGDP	LGDP^2^	LEN	LPOP
CD-test statistics	33.33^a^	92.34^a^	92.34^a^	12.00^a^	9.46^a^
*p-*value	00.00	00.00	00.00	00.00	00.00

a Rejection of *H*_*0*_ at 0.01.

#### 4.2.2 Panel unit root tests

Different results were obtained by using different stationarity tests where the null hypothesis was rejected for some variables at their first level (Table 3); however, it was rejected after taking the first difference for all series, which therefore became stationary (*p* < 0.01) (Table 3).

**Table 3:** Stationarity panel unit root tests.

Variables	Level				First Difference			
	LLC	IPS	PP-Fisher	CIPS	LLC	IPS	PP-Fisher	CIPS
LCO_2_	−2.68∗	−1.24	121.8∗	−2.50∗	−6.74∗	−12.60∗	262.6∗	−4.86∗
LGDP	−1.07	−1.31	75.59∗∗	−2.38∗	−7.20∗	−9.44∗	190.50∗	−4.40∗
LGDP^2^	−1.07	−1.31	75.59∗∗	−2.38∗	−7.20∗	−9.44∗	190.50∗	−4.40∗
LEC	−4.78∗	−3.37∗	145.99	−2.99∗	−7.13∗	−11.21∗	229.06∗	−4.61∗
LPOP	−2.12∗	−0.19	78.59∗	−1.24	−2.75∗	−3.57∗	99.31∗	−2.78∗

∗and ∗∗ significance at 0.01 and 0.05, respectively. The lag length of all unit root tests is determined using the A.I.C. Newey‒West bandwidth selection with the Bartlett Kernel is used for the L.L.C. test..

#### 4.2.3 Panel cointegration tests

In the second stage, five out of seven Pedroni test statistics were supported by the test statistics of Kao [54] and Johansen [64], rejecting the null hypothesis of no cointegration (*p* < 0.01) (Table 4), which verified the long-run cointegration relationship among the variables of equation (9) (GDP, EC, POP), in the study period (Table 4).

**Table 4:** Panel cointegration tests.

Tests	Statistics	Probability	Johansen Fisher Panel Cointegration Test		
Panel V-statistics	3.95∗∗	0.042	Number of C.E. (s)	Trace test statistics	Probability
Panel rho-statistics	1.81	0.9652	None	610.0∗	0.0000
Panel *P*P-statistics	−2.26∗∗	0.011	At most 1	283.6∗	0.0000
Panel ADF-statistics	−5.92∗	0.000	At most 2	152.8∗	0.0001
Group rho-Statistic	1.89	0.971	At most 3	97.48∗∗∗	0.0097
Group *P*P-Statistic	−7.96∗	0.000	At most 4	87.82∗∗	0.0027
Group ADF-Statistic	−5.00∗	0.000	∗ Probabilities are computed using asymptotic Chi-square distribution		
Kao (1999)	−6.96∗	0.000			

∗and ∗∗ rejection of *H*_*0*_ at 0.01 and 0.05, respectively.

In the same context, Westerlund [65] illustrated that (Gt, Ga, Pt, and Pa) statistic tests rejected the null hypothesis of no cointegration (*p* < 0.01) (Table 5), which also confirms the long-run cointegration relationship between LCO_2_ and LGDP, LGDP^2^, LEC, and LPOP despite the presence of cross-sectional dependencies.

**Table 5:** Westerlund ECM. panel cointegration tests.

Panel cointegration tests	Statistics	*p*-value
Gt	−3.141	0.000∗
Ga	−12.993	0.067∗∗∗
Pt	−15.187	0.000∗
Pa	−13.486	0.02∗∗

*H*_*0*_: no cointegration within the EU-27 series and four covariates (CO_2_ emissions, energy consumption, GDP, population); ∗, ∗∗, and ∗∗∗rejection of *H*_*0*_ at 0.01, 0.05, and 0.1 respectively..

#### 4.2.4 Long-run estimates of panel cointegration

The signs on coefficient and elasticities of carbon emission for income, income square, energy consumption, and population did not differ significantly in FOMLS, DOLS, and AMG estimation (except for POP). Moreover, due to the data's cross-sectional dependency, we considered the third-generation estimation methodology (i.e., AMG) since it is more reliable [66]. The significant positive sign on LGDP and significant negative sign on income square (LGDP^2^) indicated the existence of an EKC relation between income and CO_2_ emissions within the EU-27 countries. Thus, based on the AMG model, an increase of 1% in GDP will lead to a rise of 0.705% in environmental degradation, while a 1% increase in GDP^2^ will reduce environmental pollution by 0.062% in the long run (*p* < 0.01) (Table 6). This implies that economic development in EU-27 reached the stage where economic growth improves the environment. On the other hand, the positive sign on the coefficient of energy consumption and population growth indicates an increase in energy consumption, and population growth will increase CO_2_ emissions.

**Table 6:** Long-run estimation of panel cointegration relation.

Regressors	FMOLS		DOLS		AMG	
	Coefficient	Probability	Coefficient	Probability	Coefficient	Probability
LGDP	0.91∗	0.00	0.96∗	0.000	0.705∗∗	0.000
LGDP^2^	−0.11∗	0.00	−0.11∗	0.000	−0.062∗∗	0.000
LEC	1.17∗	0.00	1.13∗	0.000	0.748∗	0.000
LPOP	−0.11∗	0.00	−0.12∗∗	0.007	1.305∗∗	0.000

∗and ∗∗ indicate significance at 0.01 and 0.05, respectively.

## 5 Discussion and concluding remarks

EU countries participate in global trade and international agreements, necessitating policy measures considering the carbon leakage effect for GHG emission mitigation. Various laws and policies targeting sectors, such as land, energy, transport, and agriculture, are implemented at the national and EU levels (e.g., European Union's Nitrates Directive) [67]. Research by Simionescu et al. [68] showed that between 2002 and 2019, facilitating environmentally friendly technologies and renewable energy sources by laws, regulatory means, and corruption control ensured reduced GHG emissions. The provision of credit to the private sector in eastern and central EU countries did not reduce GHG emissions because of mismatched grant prioritization. Previously, Lapinskienė et al. [69] revealed that higher taxes on energy production, research, and development reduced GHG emissions in EU countries between 2006 and 2013. However, the same report indicates that the approximation of research and development impacts on GHG emissions can be problematic because of variations in the timing of conducting and implementing research findings by individual countries. Bellassen et al. [70] showed that inaccuracy and imprecise reporting of soil carbon changes threaten overall GHG emissions information. The main issues are the weak regulatory incentives to boost soil carbon monitoring and soil data is expensive and scarce. The success of land-related climate policy is affected because most soil carbon stock fluxes in European soils are not tracked, making this a significant blind spot. This implies that an additional 70 Mt CO_2_ yr^−1^ emissions are omitted from croplands, although new removals of equal magnitude could appear in grasslands and forests [70].

We revealed a significant decline in CO_2_ emissions in the EU-27 between 1990 and 2019 (Fig. 2, Fig. 3). Also, the output of this research indicates the existence of an EKC (inverted-U shaped) relation between GDP and CO_2_ emissions in the EU-27. Even though our research was conducted within 27 EU countries and POP was involved as a controlled variable, our finding aligns with previous studies, as highlighted in Table 7.

**Table 7:** An overview of the previous research related to the EKC analysis within Europe.

Variable	Time period	E.U. countries	Input variables	EKC relationship	References
Ecological footprint, CO_2_ emissions	14 European countries	1990–2014	Fossil fuels consumption, CO_2_ emissions, GDP, GDP^2^, RE, Ecological footprint	No	[90]
CO_2_ emissions	27 European countries	1990–2017	GDP, GDP^2^, energy intensity, CO_2_ emissions	Yes	[4]
CO_2_ emissions	34 European countries	1990–2021	RE, Nuclear energy, Research and Development in E.U., GDP, GDP^2^, CO_2_ emissions, energy intensity	No	[91]
CO_2_ emissions	Five European countries	1990–2015	GDP, foreign direct investment, energy innovation, air transport	Yes	[92]
GHG emissions (CO_2_, CH_4_, N_2_O)	Hungary	1985–2018	GHG emissions, EC, GDP	Yes	[3]
CO_2_ emissions	Visegrad countries	1990–2018	RE, non-RE, POP, foreign direct investment, GDP, CO_2_ emissions.	Yes	[93]
CO_2_ emissions	Portugal	1970–2016	Trade intensity, EC, GDP, CO_2_ emissions	Yes	[94]
CO_2_ emissions	BRICS Countries	1990–2015	GDP, GDP^2^, economic complexity index, RE, CO_2_ emissions	Yes	[95]
Nitrous oxide (N_2_O) emissions	Germany	1970–2012	Nitrous oxide (N_2_O) emissions, GDP, GDP^2^, agricultural land used and exports	Yes	[96]
Ecological footprint, CO_2_ emissions	France	1977–2017	Nuclear and RE, GDP, Ecological footprint, CO_2_ emissions	No	[97]

Structural changes in the economy, the use of renewable sources of energy, less carbon-intensive fuels, and improvements in energy efficiency accounted for the reductions [68,71]. Energy production and consumption are essential for economic development; therefore, they remain the key drivers of anthropogenic GHG emissions [72]. The relationship between GDP, energy, and POP variables can be explained by the “energy-GDP-population nexus,” which refers to the interdependence between these three factors. GDP positively correlates with energy consumption, as increased economic activity requires more energy [73]. Population growth also contributes to increased energy consumption, which means greater demand for energy services [74]. The population and GDP relationship is typically positive [75] where a larger population generally means a more significant labor force, which can produce more goods and services, leading to increased economic output. However, the relationship between these variables and CO_2_ emissions is complex and interdependent.

Energy consumption is one of the main drivers of CO_2_ emissions [76], particularly from burning fossil fuels. As nations' GDP increases, so does their energy demand, producing higher CO_2_ emissions [77]. In this context, the relationship is not necessarily linear due to many factors, such as the adaptation of renewable energy or implementation of energy-efficient practices in different sectors, which directly decoupled GDP and CO_2_ emissions. On the other hand, population and CO_2_ emissions have a complex interaction. However, population growth can drive innovation and technological advancements that reduce emissions.

Additionally, changes in individual behaviors and consumption patterns can significantly impact emissions, regardless of population size. However, reducing CO_2_ emissions requires addressing the interconnections between energy, GDP, and population. This can involve promoting sustainable and efficient energy practices, investing in clean energy technology, and making behavioral changes that reduce consumption and waste. The European Environment Agency (EEA) [71] revealed that it is difficult to decouple emissions from economic growth in countries whose GDP is largely based on fossil fuels. However, according to the European neutrality law, it is possible to decouple economic growth from GHG emissions by having specific inclusive sector dialogs and partnerships between stakeholders to reduce the EU's greenhouse gas emissions in a cost-effective manner, such as the use of renewable energy and enhancing removals by sinks, to achieve the climate neutrality objective by 2050. To achieve this, all citizens' and consumers' empowerment in making change possible and providing accurate information to the public is pivotal. This aligns with policy actions related to information and voluntary mitigation strategies. For example, climate change, values, rights, the rule of law, and security conferences on a European platform represent an effective information-sharing framework [68].

To achieve the GHG 2050 neutrality objective, further research on energy, technology, economic cost-benefit analysis, and proper economic and regulatory frameworks will foster the development and diffusion of technological innovations. EEA [78] reported that the reduction in GHG emissions mainly occurred among electricity, heat, and combined heat and power producers. The increased use of renewable energy sources in power generation reduced emissions by 50 million tonnes in 2018 compared to 2017. According to Simionescu et al. [68], the achievement of the 27% use of renewable energy sources as per the EU's Renewable Energy Directive by 2030 will depend on each country's level of economic development, structure of energy consumption, and policies for achieving energy efficiency. Similarly, Mielcarek-Bocheńska and Rzeźnik [79] show that technological changes in EU countries, the size of countries, and the proportion of changes in GHG emitting sectors also contribute to variations in GHG emissions. Germany's low emissions are attributed to innovative technology, research, and renewable energy sources. However, it is unclear that although Germany has the highest GHG emission reduction, it registered the highest drop in energy efficiency. Unsurprisingly, countries such as Latvia had the lowest emission reduction. Kar [80] shows that economic growth had an insignificant impact in mitigating CO_2_ emissions in Baltic countries, including Latvia. Surprisingly, these countries had not achieved the required GHG reduction limit by 2020, yet the current updated target by Fit for 55 initiatives by 2030 is much higher. This means these countries must take robust measures in emission trading and non-emission trading system sectors. Coordinated efforts, policies, and instruments benefit from enhanced synergism. In fact, GHG emission reduction requires changes in legislation, financial outlays, organization, and technical changes [79], which should not be taken in isolation but in a nexus with national and international laws because of the carbon leakage effect. However, generally, the results show that implementing GHG emissions reduction policies, such as technology and research, incentives, and economic adjustments, in countries such as Germany, France, Denmark, and the Czech Republic contribute to reducing GHG emissions. However, the extent of the impact is debatable.

Conversely, Petrović et al. [81] show that increased service sector participation in highly developed countries, such as France, Germany, Belgium, and the Netherlands, contributes to reduced transport GHG emissions. At the same time, the reverse is true for transition countries, such as Malta, Latvia, Hungary, Slovakia, and Estonia. This implies that environmental and climate benefits policies are being achieved by EU countries, although at varying levels [82] except for countries with a positive relation. Lee and van de Meene [82] define the environmental benefits of climate change as policies to decrease GHG emissions, positively impact energy consumption, enhance green space, or reduce waste generation. Similarly, Ahmad et al. [83] stress the importance of coupling innovation, green strategies, financial approaches, and environmental goals.

According to Simionescu et al. [68], the policy of providing domestic credit to the private sector reduced GHG emissions and pollution in Eastern and Central European countries from 2002 to 2019, but the specification of the legislative framework conditioning credit granting is suggested as a measure to ensure the proper execution of environmentally friendly technologies. Generally, the decreased emissions and high GDP in some EU countries imply that implementing GHG emission policies and strategies does not conflict with a growing economy [78]. According to our analysis, economic development in the EU has reached the stage where economic growth supports sustainability, as the EKC explains (Table 6). These results align with Dogan and Inglesi-Lotz [84] and Neagu [37]. Jóźwik et al. [85] confirmed the EKC hypothesis only in Poland out of all Central European countries, which implies that in the studied period, EU countries have progressed significantly toward the reduction of GHGs, suggesting that if a similar trend follows, the set target of 2030 and 2050 will be achievable. The positive sign of the coefficients of energy consumption and population growth in the FMOLS model indicates an increase in GHG emissions, in line with the findings of Dogan et al. [86].

Overall, implementing policies targeting energy production and consumption, population dynamics and structure, land, and agriculture of each country are critical to achieving EU countries' 2030 and 2050 GH G reduction targets. The results of this study show that the economic development exhibited by increased GDP reduces GHG emissions and consequently supports environmental protection. Although our results do not provide a detailed analysis of all GHG contributors per country, which may be necessary for the contextualized assessment of individual countries’ progress in achieving GHG reduction set targets, our results provide a general overview of the GHG emission trend at the EU level, a perspective that has been lacking. Consequently, our results affect several national, regional, and international stakeholders. A detailed study analyzing the contrast between the reduction in GHG emissions, and the reduced energy efficiency needs to be conducted for Germany and other individual countries to assess trends of GHG emissions, energy efficiency, economic performance, and the effectiveness of specialized national environmental policies. Overall, this study provides valuable insights for decision-makers on the European Commission, offering a robust evaluation of the direct impact of all policies on environmental deterioration between 1990 and 2019.

## CRediT authorship contribution statement

**Safwan Mohammed:** Conceptualization, Formal Analysis, Visualization, Writing - Original Draft, Writing - Review & Editing. **Abid Rashid Gill:** Formal Analysis, Writing - Original Draft. **Main Al-Dalahmeh:** Writing - Original Draft. **Ali Alkerdi:** Writing - Original Draft. **Akasairi Ocwa:** Writing - Original Draft. **Karam Alsafadi:** Visualization. **Kaushik Ghosal:** Writing - Review & Editing. **Szilárd Szabó:** Writing - Review & Editing. **Judit OlÁh:** Writing - Review & Editing. **Endre Harsanyi:** Writing - Review & Editing, Funding Acquisition.

## Declaration of competing interest

The authors declare that they have no known competing financial interests or personal relationships that could have appeared to influence the work reported in this paper.
