# Supplementary material for: Assessment of the environmental kuznets curve within EU-27: Steps toward environmental sustainability (1990–2019)
Source: Environ Sci Ecotechnol. 2023 Sep 19;18:100312. doi: 10.1016/j.ese.2023.100312 (PMC10628553; doi:10.1016/j.ese.2023.100312)

Table S1. Detailed results of Trend analysis (M.K. test and *Ss*) of studied EU-27 parameters (1990–2019)

|  | EC | | | | | CO_2_ emissions | | | | | GDP | | | | | POP | | | | |
| --- | --- | --- | --- | --- | --- | --- | --- | --- | --- | --- | --- | --- | --- | --- | --- | --- | --- | --- | --- | --- |
| **Code*** | **Tau** | **P** | **Z** | **Ss** | **BR** | **Tau** | **P** | **Z** | **Ss** | **BR** | **Tau** | **P** | **Z** | **Ss** | **BR** | **Tau** | **P** | **Z** | **Ss** | **BR** |
| **EU27** | 0.29 | 0.02 | 2.24 | 1.18 | 2000 | -0.5 | <0.001 | -3.92 | -24.24 | 2008 | 0.9 | <0.001 | 7.63 | 301.56 | 2004 | 0.986 | <0.001 | 7.63 | 1001.3 | 2004 |
| **BE** | 0.23 | 0.07 | 1.78 | 0.07 | 1996 | -0.47 | 0.0002 | -3.67 | -0.92 | 2008 | 0.99 | <0.001 | 7.67 | 10.61 | 2004 | 1 | <0.001 | 7.74 | 53.5 | 2006 |
| **BG** | -0.31 | 0.01 | -2.39 | -0.04 | 1998 | -0.59 | <0.001 | -4.56 | -0.55 | 1998 | 0.92 | <0.001 | 7.13 | 1.86 | 2005 | -1 | <0.001 | -7.74 | -60 | 2002 |
| **CZ** | -0.55 | <0.001 | -4.31 | -0.11 | 1997 | -0.78 | <0.001 | -6.06 | -1.44 | 2007 | 0.94 | <0.001 | 7.35 | 6.7 | 2004 | 0.4 | <0.001 | 3.1 | 10.64 | 2008 |
| **DK** | -0.02 | 0.83 | -0.21 | -0.006 | 2011 | -0.73 | <0.001 | -5.7 | -1.2 | 2007 | 0.99 | <0.001 | 7.67 | 6.9 | 2004 | 1 | <0.001 | 7.74 | 21.38 | 2006 |
| **DE** | -0.52 | <0.001 | -4.06 | -0.54 | 2006 | -0.88 | <0.001 | -6.81 | -7.51 | 2004 | 0.97 | <0.001 | 7.56 | 58.8 | 2005 | 0.32 | 0.01 | 2.53 | 46 | 1995 |
| **IE** | 0.56 | <0.001 | 4.35 | 0.15 | 1999 | 0.27 | 0.03 | 2.1 | 0.25 | 1998 | 0.9 | <0.001 | 7.02 | 9.75 | 2003 | 1 | <0.001 | 7.74 | 53.42 | 2005 |
| **EE** | -0.05 | 0.69 | -0.39 | -0.001 | 1992 | -0.25 | 0.05 | -1.92 | -0.12 | 1994 | 0.95 | <0.001 | 7.38 | 0.89 | 2005 | -0.92 | <0.001 | -7.08 | -6.25 | 2001 |
| **EL** | 0.2 | 0.11 | 1.57 | 0.06 | 1997 | -0.08 | 0.54 | -0.6 | -0.3 | 2011 | 0.57 | <0.001 | 4.46 | 4.45 | 2001 | 0.52 | <0.001 | 4.03 | 29.25 | 2000 |
| **ES** | 0.44 | 0.0005 | 3.46 | 0.91 | 1999 | 0.24 | 0.06 | 1.85 | 1.31 | 1998 | 0.85 | <0.001 | 6.63 | 30.53 | 2003 | 0.9 | <0.001 | 6.99 | 326.6 | 2004 |
| **FR** | 0.19 | 0.13 | 1.49 | 0.22 | 1997 | -0.42 | 0.001 | -3.24 | -2.38 | 2008 | 0.99 | <0.001 | 7.67 | 49.78 | 2004 | 1 | <0.001 | 7.74 | 341.09 | 2004 |
| **HR** | 0.47 | 0.0002 | 3.67 | 0.05 | 2002 | 0.12 | 0.33 | 0.96 | 0.05 | 2000 | 0.84 | <0.001 | 6.56 | 1.61 | 2003 | -0.82 | <0.001 | -6.35 | -19.5 | 2000 |
| **IT** | 0.24 | 0.06 | 1.85 | 0.27 | 1998 | -0.24 | 0.05 | -1.89 | -3.05 | 2008 | 0.89 | <0.001 | 6.92 | 32.92 | 2002 | 0.93 | <0.001 | 7.22 | 158.6 | 2006 |
| **CY** | 0.67 | <0.001 | 5.2 | 0.02 | 2001 | 0.51 | <0.001 | 3.99 | 0.1 | 1999 | 0.88 | <0.001 | 6.88 | 0.61 | 2003 | 0.94 | <0.001 | 7.31 | 10.39 | 2005 |
| **LV** | -0.13 | 0.3 | -1.03 | -0.007 | 1994 | -0.45 | 0.0004 | -3.49 | -0.08 | 1996 | 0.93 | <0.001 | 7.24 | 1.02 | 2005 | -1 | <0.001 | -7.74 | -26.08 | 2004 |
| **LT** | 0.18 | 0.16 | 1.39 | 0.01 | 1992 | -0.31 | 0.01 | -2.39 | -0.09 | 1993 | 0.96 | <0.001 | 7.47 | 1.62 | 2005 | -0.98 | <0.001 | -7.61 | -34.9 | 2005 |
| **LU** | 0.44 | 0.0005 | 3.46 | 0.03 | 2002 | -0.05 | 0.66 | -0.42 | -0.01 | 2002 | 0.99 | <0.001 | 7.67 | 1.78 | 2005 | 1 | <0.001 | 7.74 | 7.25 | 2006 |
| **HU** | 0.29 | 0.02 | 2.24 | 0.05 | 2002 | -0.76 | <0.001 | -5.88 | -0.72 | 2008 | 0.93 | <0.001 | 7.24 | 4.14 | 2003 | -0.99 | <0.001 | -7.7 | -23.18 | 2004 |
| **MT** | 0.66 | <0.001 | 5.17 | 0.006 | 2006 | -0.2 | 0.11 | -1.57 | -0.008 | 2014 | 0.99 | <0.001 | 7.7 | 0.28 | 2007 | 1 | <0.001 | 7.74 | 2.9 | 2006 |
| **NL** | -0.07 | 0.56 | -0.57 | -0.03 | 2013 | -0.14 | 0.25 | -1.14 | -0.19 | 2010 | 0.99 | <0.001 | 7.67 | 18.5 | 2003 | 1 | <0.001 | 7.74 | 77.05 | 2002 |
| **AT** | 0.79 | <0.001 | 6.13 | 0.28 | 2001 | 0.3 | 0.01 | 2.35 | 0.21 | 2000 | 0.99 | <0.001 | 7.7 | 8.66 | 2005 | 1 | <0.001 | 7.74 | 32.25 | 2005 |
| **PL** | 0.42 | 0.001 | 3.24 | 0.32 | 2007 | -0.38 | 0.002 | -2.99 | -1.58 | 1998 | 0.95 | <0.001 | 7.42 | 16.42 | 2005 | -0.66 | <0.001 | -5.1 | -17.64 | 2002 |
| **PT** | 0.31 | 0.01 | 2.39 | 0.14 | 1998 | 0.011 | 0.94 | 0.07 | 0.01 | 1997 | 0.9 | <0.001 | 7.02 | 5.02 | 2002 | 0.5 | <0.001 | 3.9 | 19.66 | 2000 |
| **RO** | -0.45 | 0.0004 | -3.53 | -0.15 | 1998 | -0.75 | <0.001 | -5.82 | -2.28 | 1998 | 0.94 | <0.001 | 7.35 | 6.94 | 2005 | -1 | <0.001 | -7.7 | -140.5 | 2005 |
| **SI** | 0.54 | <0.001 | 4.21 | 0.04 | 2000 | 0.029 | 0.83 | 0.21 | 0.009 | 2012 | 0.93 | <0.001 | 7.24 | 1.3 | 2004 | 0.71 | <0.001 | 5.48 | 3.14 | 2008 |
| **SK** | -0.4 | 0.001 | -3.1 | -0.04 | 2008 | -0.82 | <0.001 | -6.35 | -0.5 | 2006 | 0.97 | <0.001 | 7.52 | 3.12 | 2005 | 0.74 | <0.001 | 5.71 | 4.45 | 1997 |
| **FI** | 0.52 | <0.001 | 4.03 | 0.13 | 2000 | -0.29 | 0.02 | -2.24 | -0.44 | 2010 | 0.93 | <0.001 | 7.24 | 5.57 | 2004 | 1 | <0.001 | 7.74 | 18.6 | 2005 |
| **SE** | -0.34 | 0.007 | -2.67 | -0.07 | 2004 | -0.78 | <0.001 | -6 | -0.59 | 2007 | 0.91 | <0.001 | 7.06 | 11.34 | 2005 | 1 | <0.001 | 7.74 | 49.28 | 2007 |

*Abbreviation for the Member States of the European Union (EU) as suggested by: <https://ec.europa.eu/eurostat/statistics-explained/index.php?title=Glossary:Country_codes> (Belgium (BE), Bulgaria (BG), Czechia (CZ), Denmark (DK), Germany (DE), Estonia (EE), Ireland (IE), Greece (EL), Spain (ES), France (FR), Croatia (HR), Italy (IT), Cyprus (CY), Latvia (LV), Lithuania (LT), Luxembourg (LU), Hungary (HU), Malta (MT), Netherlands (NL), Austria (AT), Poland (PL), Portugal (PT), Romania (RO), Slovenia (SI), Slovakia (SK), Finland (FI), Sweden (SE))

**Figure. S1.** Trend analysis (M.K. test & Ss) of studied EU-27 parameters (1990-2019): a) energy consumption, b) CO_2_ emission, c) GDP, and d) population (POP) (▲: positive trend, ▼negative trend, ○ significant)


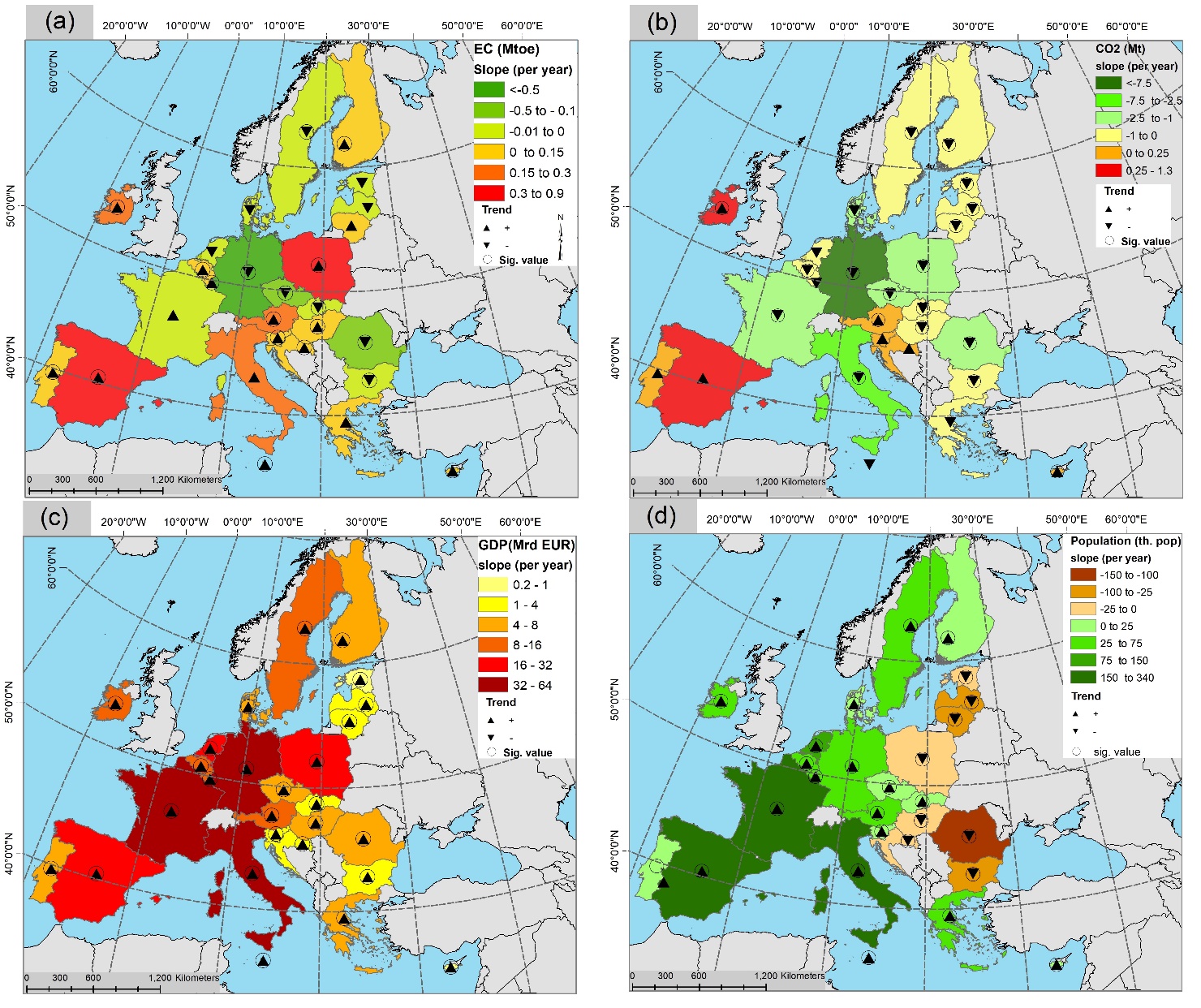

Supplement: Multimedia component 1 [file mmc1.docx]
